# Crystal structure and Hirshfeld surface analysis of chlorido­(2,6-di­methyl­phenyl isocyanide)[*N*′-(2,6-di­methyl­phen­yl)-*N*-(pyridin-2-yl)carbamimido­yl]platinum(II)

**DOI:** 10.1107/S2056989025005079

**Published:** 2025-06-10

**Authors:** Olga V. Repina, Elena Yu. Nevskaya, Ilya S. Kritchenkov, Victor N. Khrustalev, Alexander S. Novikov, Alexander G. Tskhovrebov, Namiq Q. Shikhaliyev, Aytan A. Niyazova, Mehmet Akkurt, Ajaya Bhattarai

**Affiliations:** a Peoples’ Friendship University of Russia, 6 Miklukho-Maklaya Street, Moscow, 117198, Russian Federation; b Zelinsky Institute of Organic Chemistry Russian Academy of Sciences (RAS) Leninsky Prospect 47 119991 Moscow Russian Federation; c Institute of Chemistry, Saint Petersburg State University, Universitetskaya Nab. 7/9, 199034 Saint Petersburg, Russian Federation; d Baku Engineering University, Khirdalan City, 120 AZ0101 Hasan Aliyev Street, Baku, Azerbaijan; e Azerbaijan State University of Economics, M. Mukhtarov 194, 1001 Baku, Azerbaijan; f Department of Physics, Faculty of Sciences, Erciyes University, 38039 Kayseri, Türkiye; g Department of Chemistry, M.M.A.M.C (Tribhuvan University) Biratnagar, Nepal; University of Neuchâtel, Switzerland

**Keywords:** crystal structure, dimers, square-planar geometry, isocyanides, 2-amino­pyridine, Hirshfeld surface analysis

## Abstract

In the title compound, [Pt(C_14_H_14_N_3_)Cl(C_9_H_9_N)], the platinum atom has a square-planar geometry. In the crystal, dimers with *R*^2^_2_(8) motifs are formed by pairs of N—H⋯N hydrogen bonds. They are connected to each other through pairs of weak C—H⋯Cl inter­actions, forming a *R*^2^_2_(16) motif, creating parallel ribbons along the [011] axis. The mol­ecular pairs are also linked by C—H⋯π and by π–π inter­actions, the shortest centroid-centroid distance [3.513 (2) Å] being observed between pyridyl rings. These weak inter­actions form parallel ribbons along the [010] axis. The resulting three-dimensional network ensures the cohesion of the crystal structure. Hirshfeld two-dimensional fingerprint plots revealed that the most significant inter­actions are H⋯H (58.0%), C⋯H/H⋯C (17.9%), Cl⋯H/H⋯Cl (10.7%), N⋯H/H⋯N (6.9%), C⋯C (2.9%), Pt⋯H/H⋯Pt (2.6%), and N⋯C/C⋯N (1.1%) contacts.

## Abstract 2

The coordination geometry around the platinum atom is square-planar. In the crystal, dimers with *R*^2^_2_(8) motifs, formed by pairs of N—H⋯N hydrogen bonds, are connect to each other through pairs of weak C—H⋯Cl inter­actions, forming a *R*^2^_2_(16) motif and creating parallel ribbons along the [011] axis direction. The mol­ecular pairs are connected by C—H⋯π and π–π inter­actions, forming parallel ribbons along the *b*-axis direction.

## 1. Chemical context

Acyclic di­amino­carbenes (ADCs) are well-known for their strong σ-donating properties and their ability to coordinate to metals through the carbene C atom (Alder *et al.*, 1996 ▸; Tskhovrebov *et al.*, 2012 ▸, 2013 ▸, 2018 ▸; Luzyanin *et al.*, 2009 ▸; Repina *et al.*, 2025 ▸). Among the various methods for synthesizing *N*-acyclic carbene (NAC) metal complexes, the addition of amines to the activated CN triple bond of isocyanide ligands stands out, due to its simplicity and the versatility of ligands that can be accessed (Michelin *et al.*, 2001 ▸).

*N*-Acyclic carbene complexes of platinum are of significant inter­est in applied science because of their potential applications in catalysis and photoluminescent materials and as anti­cancer agents (Kinzhalov & Luzyanin, 2022 ▸). For instance, palladium NAC complexes have been successfully utilized as Suzuki–Miyaura, Sonogashira, and Heck reaction catalysts (Mizoroki *et al.*, 1971 ▸; Heck & Nolley, 1972 ▸; Miyaura *et al.*, 1979 ▸; Suzuki, 1999 ▸). The ability of the Pd center to promote coupling between coordinated isocyanides and 2-amino­pyridine was demonstrated earlier (Tskhovrebov *et al.*, 2011 ▸; Mikhaylov *et al.*, 2020 ▸). In our study, we aimed to adapt this synthetic approach to platinum.
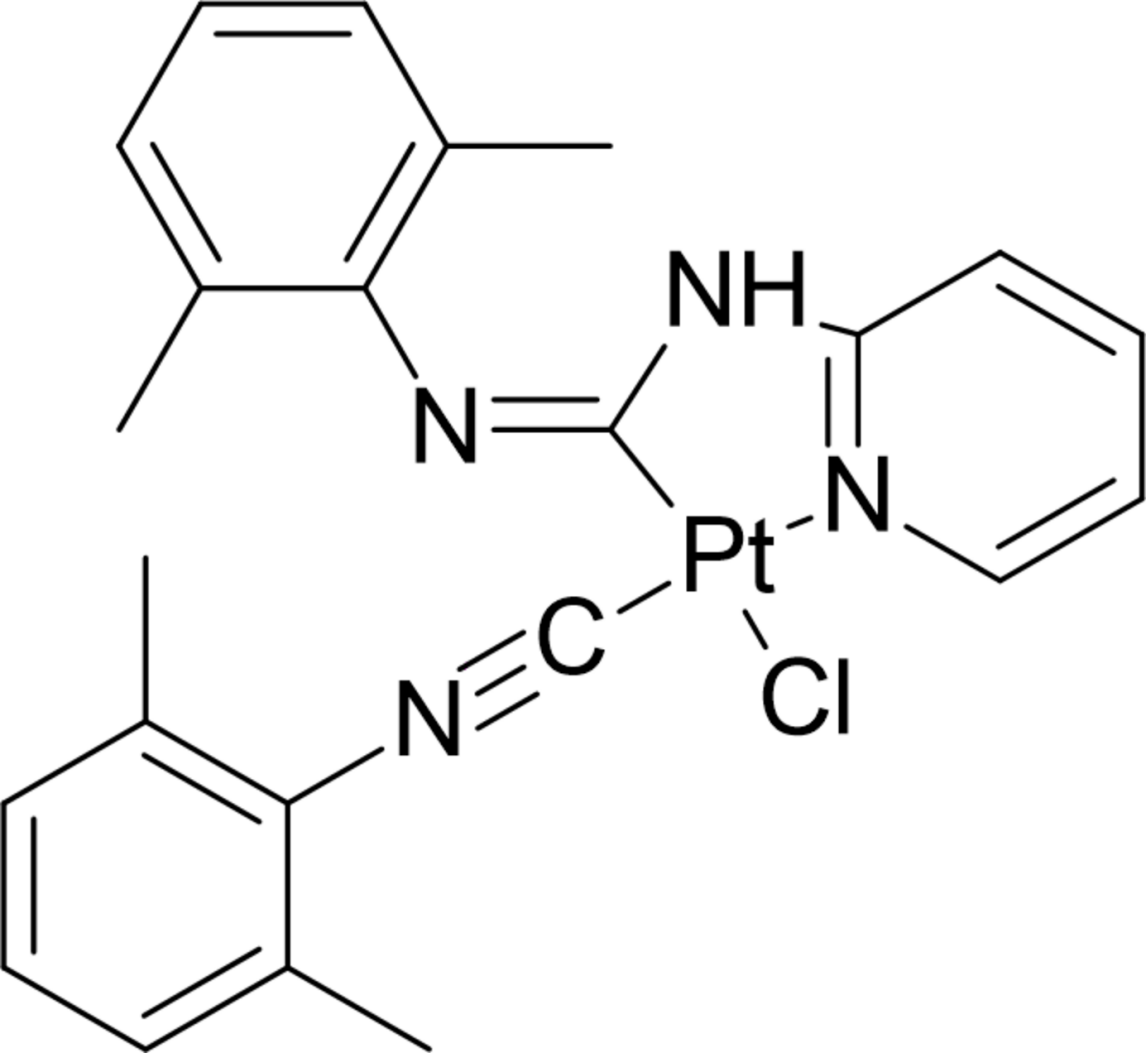


## 2. Structural commentary

**Figure 1:**
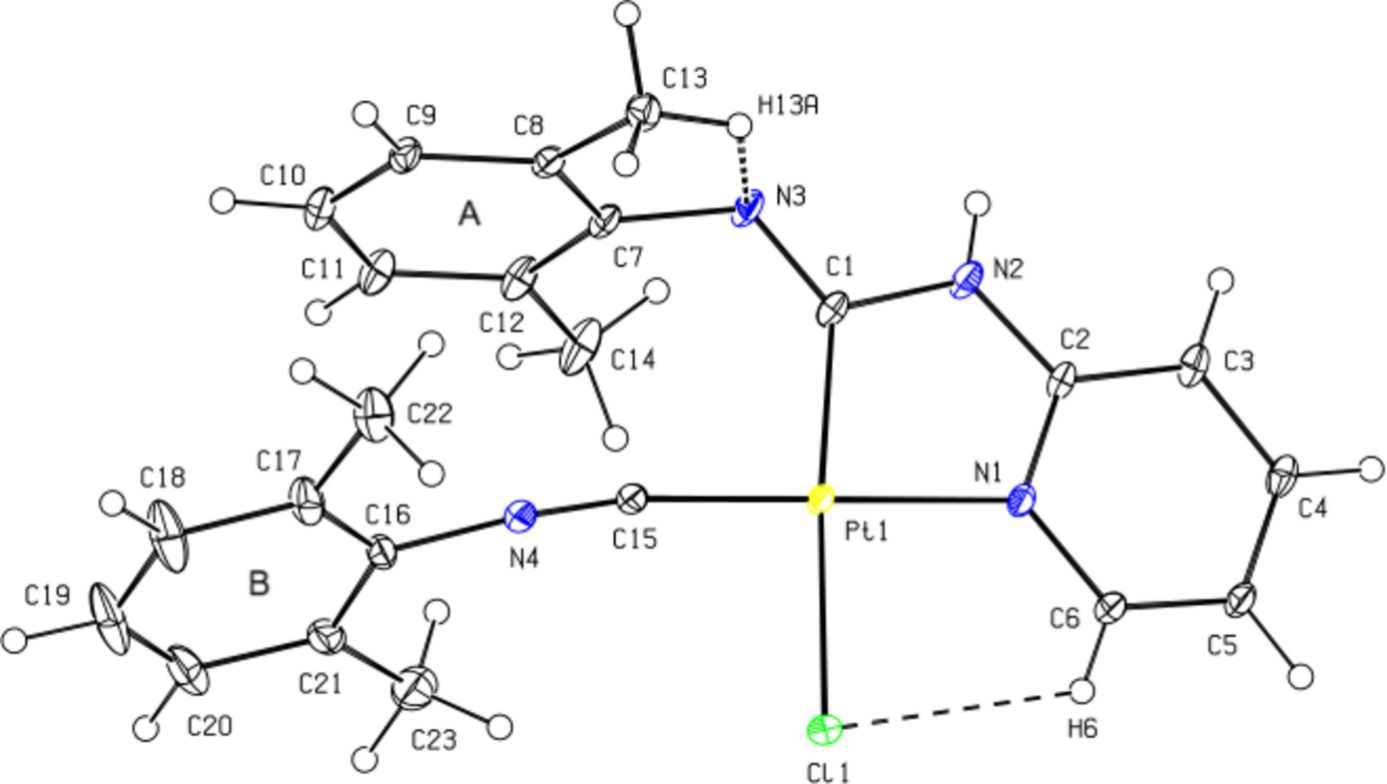
Mol­ecular structure of the title complex with displacement ellipsoids drawn at the 30% probability level.

The bond lengths involving Pt are: Pt1—C15 = 1.910 (3), Pt1—C1 = 1.979 (3), Pt1—N1 = 2.048 (3) and Pt1—Cl1 = 2.3709 (9) Å, while the sum of the angles around the Pt atom [C1—Pt1—N1 = 80.71(12°), C15—Pt1—C1 = 98.97 (14)°, C15–Pt1—Cl1 = 85.69 (10) and N1—Pt1—Cl1 = 94.63 (9)°] is 360 (14)°, thus showing a typical square-planar geometry (Fig. 1 ▸). The Pt—C_carbene_ distance [1.910 (3) Å] is slightly shorter than Pt—C_amine_ [1.979 (3) Å]. The 2,6-di­methyl­phenyl fragments (*A*: C7–C12 and *B*: C16–C21) are almost perpendicular to the mean plane passing through the square-planar complex and the pyridyl-carbamimidoyl moiety, the inter­planar angles being 81.55 (9) and 72.64 (11)°, respectively. The compound exhibits weak intra­molecular C6—H6⋯Cl1 and C13—H13*A*⋯N3 inter­actions (Fig. 1 ▸; Table 1 ▸). The geometric parameters are normal and consistent with those of related compounds (see *Database survey* section).

## 3. Supra­molecular features and Hirshfeld surface analysis

**Figure 2:**
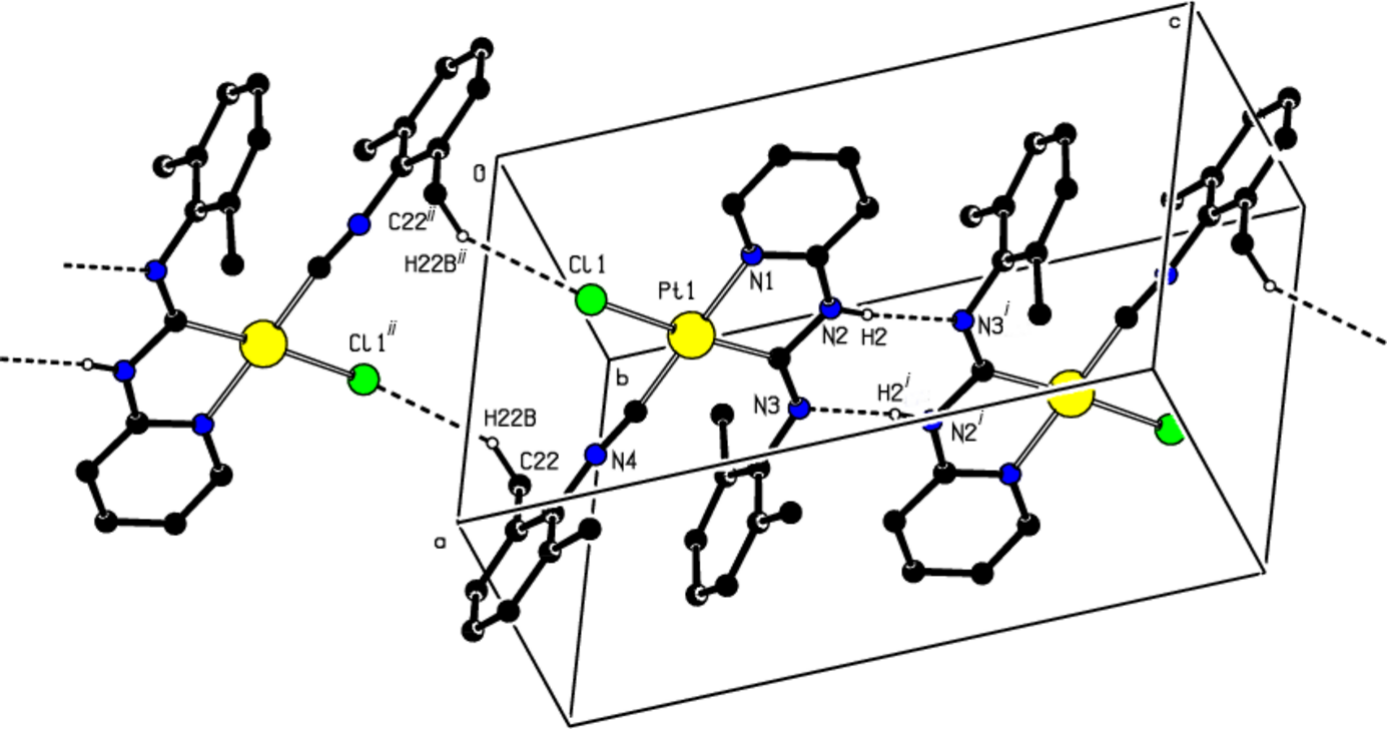
Hydrogen bonds (dotted lines) in the crystal, showing the N—H⋯N dimer and the C—H⋯Cl inter­actions.

**Figure 3:**
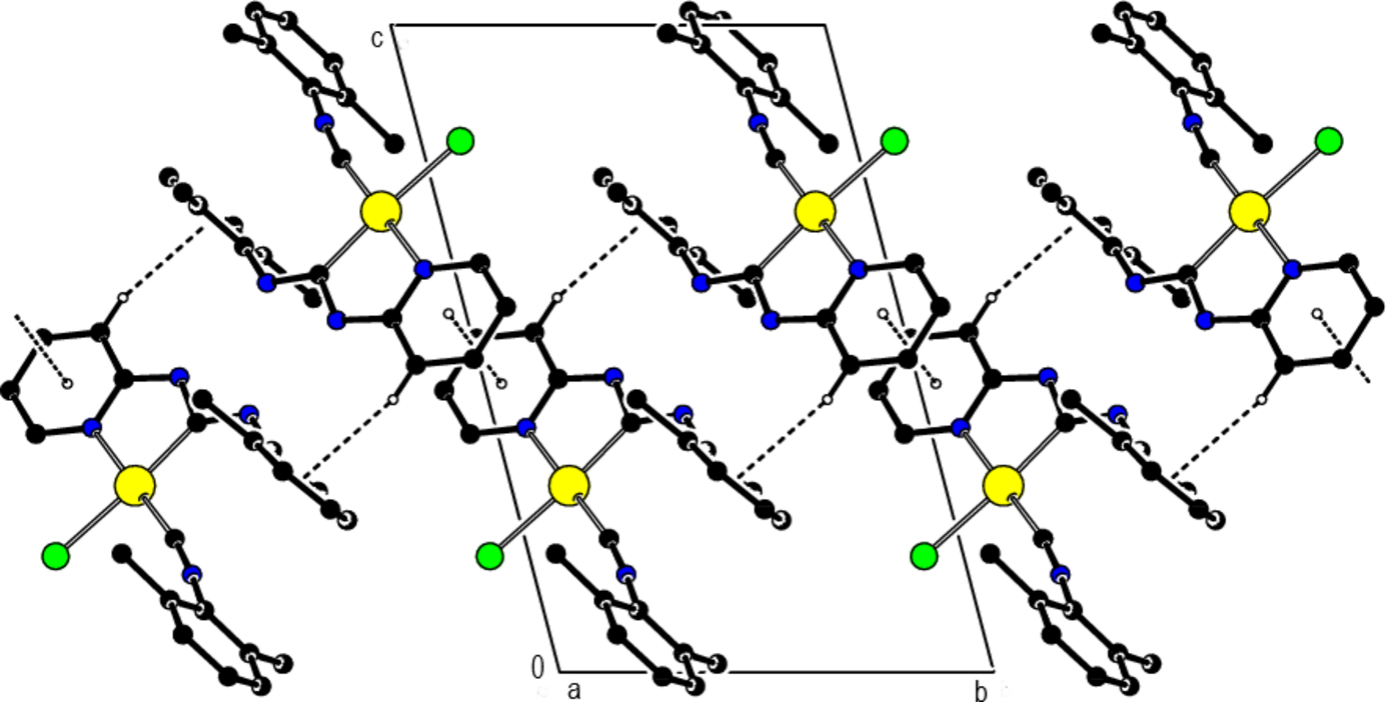
A view along the *a*-axis showing the C—H⋯π inter­actions (dotted lines).

In the crystal, mol­ecules are linked by pairs of N—H⋯N hydrogen bonds, forming inversion dimers with an 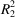
(8) ring motif (Bernstein *et al.*, 1995 ▸). The dimers are connected through pairs of weak C—H⋯Cl inter­actions, forming an 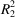
(16) motif. Thus, parallel ribbons are formed along the [011] axis (Fig. 2 ▸; Table 1 ▸). Furthermore, the mol­ecular pairs are also linked by C—H⋯π and π–π inter­actions [*Cg*2⋯*Cg*2^*a*^ = 3.513 (2) Å, slippage = 0.106 Å; symmetry code (*a*): 1 − *x*, −*y*, 1 − *z*; where *Cg*2 is the centroid of the N1/C2–C6 pyridine ring], forming parallel ribbons along the [010] axis (Table 1 ▸; Fig. 3 ▸). The three-dimensional network arising from N—H⋯N and C—H⋯Cl hydrogen bonds, C—H⋯π and π—π inter­actions, contribute to the cohesion of the crystal structure. Table 2 ▸ lists additional inter­molecular hydrogen contacts.

**Figure 4:**
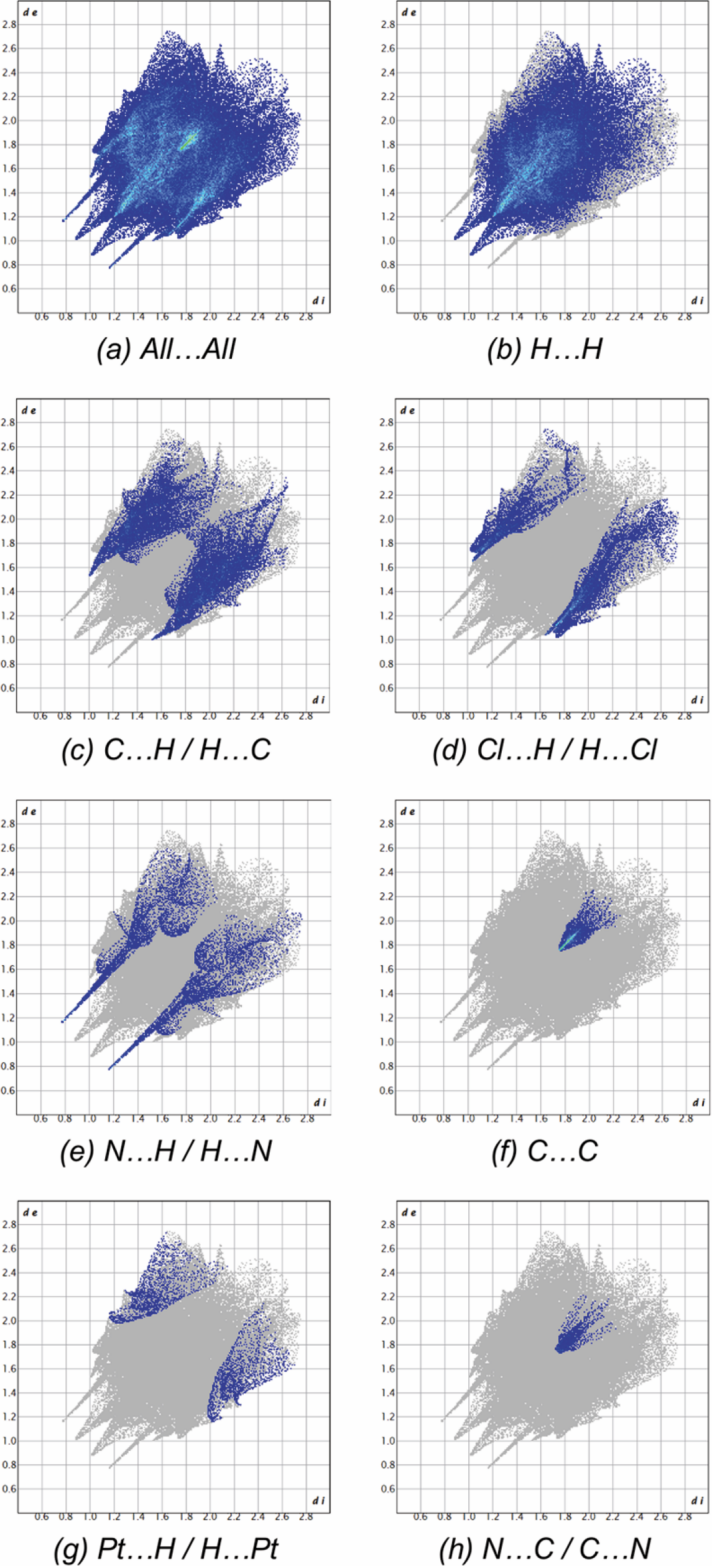
Two-dimensional fingerprint plots from a Hirshfeld surface analysis of the complex showing: (*a*) all contacts, (*b*) H⋯H (58.0%), (*c*) C⋯H/H⋯C (17.9%), (*d*) Cl⋯H/H⋯Cl (10.7%), (*e*) N⋯H/H⋯N (6.9%), (*f*) C⋯C (2.9%), (*g*) Pt⋯H/H⋯Pt (2.6%), (*h*) N⋯C/C⋯N (1.1%).

In order to qu­antify the inter­molecular inter­actions in the crystal, *Crystal Explorer 17.5* (Spackman *et al.*, 2021 ▸) was used to generate Hirshfeld surfaces and two-dimensional fingerprint plots. The contributions of different inter­molecular contacts to the Hirshfeld surface (Fig. 4 ▸) are following: H⋯H (58.0%; Fig. 4 ▸*b*), C⋯H/H⋯C (17.9%; Fig. 4 ▸*c*), Cl⋯H/H⋯Cl (10.7%; Fig. 4 ▸*d*), N⋯H/H⋯N (6.9%; Fig. 4 ▸*e*), C⋯C (2.9%; Fig. 4 ▸*f*), Pt⋯H/H⋯Pt (2.6%; Fig. 4 ▸*g*), and N⋯C/C⋯N (1.1%; Fig. 4 ▸*h*).

## 4. Database survey

A search of the Cambridge Structural Database (CSD, Version 5.44. last update Jun 2023; Groom *et al.*, 2016 ▸) for the complex yielded four closely related entries, *viz*. CSD refcodes ROJWOD (Luzyanin *et al.*, 2008 ▸), ROJWUJ (Luzyanin *et al.*, 2008 ▸), ROJXAQ (Luzyanin *et al.*, 2008 ▸), and MUDJAZ (Mikhaylov *et al.*, 2020 ▸). ROJWOD and ROJWUJ crystallize in the triclinic *P*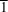 space group like the title complex, ROJXAQ in the monoclinic *C*2/c, and MUDJAZ in the monoclinic *P*2_1_/*c* space group. In ROJWOD and MUDJAZ, the Pt^II^ atom has a square-planar geometry. In the crystals of ROJWUJ and ROJXAQ, both Pt^II^ centers exhibit a slightly distorted square-planar geometry and they have the same coordination environment. In MUDJAZ, the carbene and unreacted isocyanide ligands were located in mutually *trans* positions. Such an arrangement was unexpected since it did not follow the *trans* effect rule (Shaw *et al.*, 2009 ▸). Consolidation of the unfavorable isomer was rationalized by intra-mol­ecular hydrogen bonds.

## 5. Synthesis and crystallization

**Figure 5:**
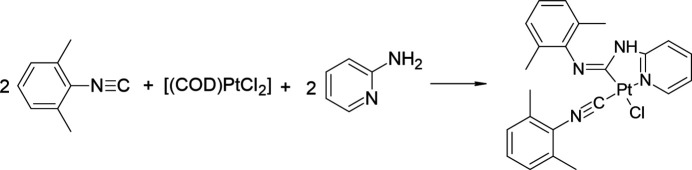
Synthesis of the title *N*-acyclic carbene complex of platinum(II).

The title compound was prepared according to a modified literature procedure (Fig. 5 ▸; Gee *et al.*, 2018 ▸). A solution of 2,6-di­methyl­phenyl­isocyanide (14 mg, 107 µmol) in 1,2-di­chloro­ethane (2 mL) was added to a solution of di­chloro (1,5-cyclo­octa­diene) platinum(II) (20 mg, 53 µmol) in 1,2-di­chloro­ethane (2 mL), and the reaction mixture was stirred at room temperature for 30 minutes. After that, 2-amino­pyridine (10 mg, 107 µmol) was added and the reaction mixture was stirred under reflux for 1 h. The course of the reaction was controlled by TLC. The solution was evaporated and the residue was purified by column chromatography (eluent: DCM), then evaporated and dried under vacuum. Yellow solid. Yield: 17 mg (55%). Crystals suitable for X-ray analysis were obtained by layering hexane over a di­chloro­methane solution of the target complex.

## 6. Refinement

Crystal data, data collection and structure refinement details are summarized in Table 3 ▸. The N-bound H atom was located in a difference-Fourier map [N2—H2 = 0.80 (6) Å] and refined with *U*_iso_(H) =1.2*U*_eq_(N). The C-bound H atoms were positioned geometrically (C—H= 0.93–0.98 Å) and refined as riding with fixed isotropic displacement parameters [*U*_iso_(H) = 1.2 or 1.5*U*_eq_(C)]. One of the methyl groups (C13) was found to be disordered; it was treated as an idealized disordered methyl group, with two positions rotated from each other by 60°, and the site-occupation factors were fixed at 0.5. Twelve reflections (−7 3 0, −8 4 0, −7 4 0, 7 − 6 1, −9 2 1, 5 − 7 2, 4 − 9 3, 5 − 9 3, −10 0 2, −11 − 2 3, 6 − 9 1 and −9 0 2) were omitted during refinement as they showed poor agreement. The remaining positive and negative residual electron densities are located near the platinum atom Pt1 (1.05 Å from Pt1) and the hydrogen atom H13*D* (0.88 Å from H13*D*), respectively.

## Supplementary Material

Crystal structure: contains datablock(s) I. DOI: 10.1107/S2056989025005079/tx2098sup1.cif

Structure factors: contains datablock(s) I. DOI: 10.1107/S2056989025005079/tx2098Isup2.hkl

CCDC reference: 2456370

Additional supporting information:  crystallographic information; 3D view; checkCIF report

## Figures and Tables

**Table 1:** Hydrogen-bond geometry (Å, °) *Cg*3 is the centroid of the C7–C12 benzene ring attached to the N3 atom.

*D*—H⋯*A*	*D*—H	H⋯*A*	*D*⋯*A*	*D*—H⋯*A*
N2—H2⋯N3^i^	0.80 (6)	2.15 (6)	2.943 (4)	173 (6)
C6—H6⋯Cl1	0.95	2.70	3.308 (4)	123
C13—H13*A*⋯N3	0.98	2.36	2.848 (5)	110
C22—H22*B*⋯Cl1^ii^	0.98	2.78	3.660 (5)	150
C3—H3⋯*Cg*3^i^	0.95	2.99	3.898 (4)	162

Symmetry codes: (i) 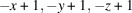; (ii) 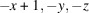.

**Table 2:** Inter­atomic contacts (Å)

Contact	Distance	Symmetry operation
H6⋯H13*B*	2.09	*x*, −1 + *y*, *z*
H13*A*⋯H11	2.32	−1 + *x*, *y*, *z*
H19⋯Cl1	2.96	2 − *x*, −*y*, −*z*
H22*B*⋯Cl1	2.78	1 − *x*, −*y*, −*z*
H2⋯N3	2.15	1 − *x*, 1 − *y*, 1 − *z*
H14*B*⋯H4	2.59	1 − *x*, −*y*, 1 − *z*
H4⋯C4	2.98	−*x*, −*y*, 1 − *z*
H18⋯H10	2.46	2 − *x*, 1 − *y*, −*z*
H14*C*⋯C14	2.94	2 − *x*, 1 − *y*, 1 − *z*

**Table 3:** Experimental details

Crystal data	
Chemical formula	[Pt(C_14_H_14_N_3_)Cl(C_9_H_9_N)]
*M*_r_	585.99
Crystal system, space group	Triclinic, *P* 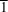
Temperature (K)	100
*a*, *b*, *c* (Å)	7.7053 (3), 9.7010 (3), 15.0316 (5)
α, β, γ (°)	104.535 (1), 95.922 (1), 90.227 (1)
*V* (Å^3^)	1081.31 (6)
*Z*	2
Radiation type	Mo *K*α
μ (mm^−1^)	6.63
Crystal size (mm)	0.20 × 0.15 × 0.10

Data collection	
Diffractometer	Bruker D8 QUEST PHOTON-III CCD
Absorption correction	Multi-scan (*SADABS*; Krause *et al.*, 2015 ▸).
*T*_min_, *T*_max_	0.297, 0.495
No. of measured, independent and observed [*I* > 2σ(*I*)] reflections	26083, 7860, 6973
*R*_int_	0.036
(sin θ/λ)_max_ (Å^−1^)	0.758

Refinement	
*R*[*F*^2^ > 2σ(*F*^2^)], *wR*(*F*^2^), *S*	0.030, 0.071, 1.09
No. of reflections	7860
No. of parameters	268
H-atom treatment	H atoms treated by a mixture of independent and constrained refinement
Δρ_max_, Δρ_min_ (e Å^−3^)	2.31, −1.84

Computer programs: *APEX3* (Bruker, 2018 ▸), *SAINT* (Bruker, 2013 ▸), *SHELXT2016/6* (Sheldrick, 2015*a* ▸), *SHELXL2016/6* (Sheldrick, 2015*b* ▸), *ORTEP-3 for Windows* (Farrugia, 2012 ▸) and *PLATON* (Spek, 2020 ▸).

## supplementary crystallographic information

### Chlorido(2,6-dimethylphenyl isocyanide)[N'-(2,6-dimethylphenyl)-N-(pyridin-2-yl)carbamimidoyl]platinum(II) Crystal data

**Table:**

[Pt(C_14_H_14_N_3_)Cl(C_9_H_9_N)]	*Z* = 2
*M**_r_* = 585.99	*F*(000) = 568
Triclinic, *P*1	*D*_x_ = 1.800 Mg m^−^^3^
*a* = 7.7053 (3) Å	Mo *K*α radiation, λ = 0.71073 Å
*b* = 9.7010 (3) Å	Cell parameters from 9990 reflections
*c* = 15.0316 (5) Å	θ = 2.7–32.6°
α = 104.535 (1)°	µ = 6.63 mm^−^^1^
β = 95.922 (1)°	*T* = 100 K
γ = 90.227 (1)°	Prism, red
*V* = 1081.31 (6) Å^3^	0.20 × 0.15 × 0.10 mm

### Chlorido(2,6-dimethylphenyl isocyanide)[N'-(2,6-dimethylphenyl)-N-(pyridin-2-yl)carbamimidoyl]platinum(II) Data collection

**Table:**

Bruker D8 QUEST PHOTON-III CCD diffractometer	6973 reflections with *I* > 2σ(*I*)
φ and ω scans	*R*_int_ = 0.036
Absorption correction: multi-scan (SADABS; Krause *et al.*, 2015).	θ_max_ = 32.6°, θ_min_ = 2.3°
*T*_min_ = 0.297, *T*_max_ = 0.495	*h* = −11→11
26083 measured reflections	*k* = −14→14
7860 independent reflections	*l* = −22→22

### Chlorido(2,6-dimethylphenyl isocyanide)[N'-(2,6-dimethylphenyl)-N-(pyridin-2-yl)carbamimidoyl]platinum(II) Refinement

**Table:**

Refinement on *F*^2^	Primary atom site location: difference Fourier map
Least-squares matrix: full	Secondary atom site location: difference Fourier map
*R*[*F*^2^ > 2σ(*F*^2^)] = 0.030	Hydrogen site location: mixed
*wR*(*F*^2^) = 0.071	H atoms treated by a mixture of independent and constrained refinement
*S* = 1.09	*w* = 1/[σ^2^(*F*_o_^2^) + (0.0282*P*)^2^ + 2.7164*P*] where *P* = (*F*_o_^2^ + 2*F*_c_^2^)/3
7860 reflections	(Δ/σ)_max_ = 0.002
268 parameters	Δρ_max_ = 2.31 e Å^−^^3^
0 restraints	Δρ_min_ = −1.84 e Å^−^^3^

### Chlorido(2,6-dimethylphenyl isocyanide)[N'-(2,6-dimethylphenyl)-N-(pyridin-2-yl)carbamimidoyl]platinum(II) Special details

**Table:**

Geometry. All esds (except the esd in the dihedral angle between two l.s. planes) are estimated using the full covariance matrix. The cell esds are taken into account individually in the estimation of esds in distances, angles and torsion angles; correlations between esds in cell parameters are only used when they are defined by crystal symmetry. An approximate (isotropic) treatment of cell esds is used for estimating esds involving l.s. planes.

### Chlorido(2,6-dimethylphenyl isocyanide)[N'-(2,6-dimethylphenyl)-N-(pyridin-2-yl)carbamimidoyl]platinum(II) Fractional atomic coordinates and isotropic or equivalent isotropic displacement parameters (Å^2^)

**Table:**

	*x*	*y*	*z*	*U*_iso_*/*U*_eq_	Occ. (<1)
Pt1	0.53441 (2)	0.13401 (2)	0.28812 (2)	0.01514 (4)	
Cl1	0.51685 (14)	−0.09097 (9)	0.17919 (8)	0.0293 (2)	
N1	0.3881 (4)	0.0699 (3)	0.3776 (2)	0.0167 (5)	
N2	0.4380 (4)	0.3015 (3)	0.4560 (2)	0.0208 (6)	
N3	0.6105 (4)	0.4395 (3)	0.3983 (2)	0.0205 (6)	
N4	0.7598 (4)	0.2117 (3)	0.1508 (2)	0.0194 (6)	
C1	0.5383 (4)	0.3147 (4)	0.3855 (2)	0.0167 (6)	
C2	0.3667 (4)	0.1731 (4)	0.4533 (2)	0.0169 (6)	
C3	0.2794 (5)	0.1459 (4)	0.5250 (3)	0.0204 (7)	
H3	0.265681	0.219578	0.578710	0.024*	
C4	0.2142 (5)	0.0100 (4)	0.5158 (3)	0.0215 (7)	
H4	0.156299	−0.011383	0.563766	0.026*	
C5	0.2336 (5)	−0.0964 (4)	0.4353 (3)	0.0240 (8)	
H5	0.187654	−0.190204	0.427571	0.029*	
C6	0.3199 (5)	−0.0627 (4)	0.3681 (3)	0.0226 (7)	
H6	0.332495	−0.134323	0.313232	0.027*	
C7	0.7334 (5)	0.4707 (3)	0.3421 (2)	0.0176 (6)	
C8	0.6827 (5)	0.5520 (3)	0.2788 (2)	0.0175 (6)	
C9	0.8120 (5)	0.6020 (4)	0.2349 (2)	0.0200 (7)	
H9	0.780448	0.658775	0.192975	0.024*	
C10	0.9859 (5)	0.5697 (4)	0.2517 (3)	0.0237 (7)	
H10	1.072772	0.606706	0.222696	0.028*	
C11	1.0329 (5)	0.4835 (4)	0.3108 (3)	0.0256 (8)	
H11	1.151099	0.457850	0.319630	0.031*	
C12	0.9076 (5)	0.4341 (4)	0.3573 (3)	0.0231 (7)	
C13	0.4921 (5)	0.5789 (4)	0.2586 (3)	0.0238 (7)	
H13A	0.423489	0.535280	0.296317	0.036*	0.5
H13B	0.474198	0.681757	0.273333	0.036*	0.5
H13C	0.454504	0.537103	0.192970	0.036*	0.5
H13D	0.477972	0.634147	0.212097	0.036*	0.5
H13E	0.427263	0.487670	0.235081	0.036*	0.5
H13F	0.446956	0.632323	0.315444	0.036*	0.5
C14	0.9594 (6)	0.3416 (5)	0.4217 (3)	0.0335 (10)	
H14A	1.086152	0.349498	0.438079	0.050*	
H14B	0.925098	0.242238	0.391146	0.050*	
H14C	0.900736	0.372707	0.477880	0.050*	
C15	0.6750 (4)	0.1938 (3)	0.2063 (3)	0.0182 (6)	
C16	0.8932 (5)	0.2184 (4)	0.0958 (3)	0.0223 (7)	
C17	0.8685 (5)	0.2968 (5)	0.0294 (3)	0.0268 (8)	
C18	1.0081 (7)	0.3038 (7)	−0.0215 (3)	0.0468 (14)	
H18	0.998231	0.356724	−0.066964	0.056*	
C19	1.1612 (7)	0.2343 (8)	−0.0066 (3)	0.0505 (15)	
H19	1.255256	0.241513	−0.041498	0.061*	
C20	1.1796 (6)	0.1548 (6)	0.0582 (3)	0.0365 (11)	
H20	1.284531	0.106110	0.066206	0.044*	
C21	1.0446 (5)	0.1458 (4)	0.1116 (3)	0.0267 (8)	
C22	0.6990 (6)	0.3677 (5)	0.0129 (3)	0.0314 (9)	
H22A	0.661132	0.418583	0.072357	0.047*	
H22B	0.609921	0.295356	−0.019962	0.047*	
H22C	0.715665	0.435391	−0.024357	0.047*	
C23	1.0591 (6)	0.0627 (5)	0.1833 (4)	0.0386 (11)	
H23A	1.162807	0.004344	0.176843	0.058*	
H23B	0.954809	0.000691	0.175119	0.058*	
H23C	1.069049	0.128459	0.244974	0.058*	
H2	0.434 (8)	0.372 (6)	0.497 (4)	0.046*	

### Chlorido(2,6-dimethylphenyl isocyanide)[N'-(2,6-dimethylphenyl)-N-(pyridin-2-yl)carbamimidoyl]platinum(II) Atomic displacement parameters (Å^2^)

**Table:**

	*U*^11^	*U*^22^	*U*^33^	*U*^12^	*U*^13^	*U*^23^
Pt1	0.01269 (5)	0.01191 (5)	0.02260 (7)	−0.00027 (4)	0.00389 (4)	0.00688 (4)
Cl1	0.0335 (5)	0.0139 (3)	0.0412 (6)	−0.0005 (3)	0.0197 (4)	0.0019 (3)
N1	0.0123 (12)	0.0144 (12)	0.0263 (15)	0.0002 (10)	0.0036 (11)	0.0097 (11)
N2	0.0271 (15)	0.0177 (13)	0.0174 (14)	−0.0097 (12)	0.0016 (12)	0.0046 (11)
N3	0.0285 (15)	0.0171 (13)	0.0179 (14)	−0.0074 (11)	0.0034 (12)	0.0077 (11)
N4	0.0175 (13)	0.0155 (12)	0.0247 (15)	−0.0017 (10)	0.0043 (11)	0.0030 (11)
C1	0.0181 (15)	0.0157 (14)	0.0176 (15)	−0.0041 (11)	−0.0017 (12)	0.0082 (12)
C2	0.0158 (14)	0.0186 (14)	0.0178 (15)	−0.0061 (11)	−0.0010 (12)	0.0085 (12)
C3	0.0204 (16)	0.0249 (16)	0.0177 (16)	−0.0078 (13)	−0.0011 (12)	0.0101 (13)
C4	0.0199 (16)	0.0233 (16)	0.0264 (18)	−0.0028 (13)	0.0016 (13)	0.0159 (14)
C5	0.0199 (16)	0.0153 (14)	0.041 (2)	−0.0001 (12)	0.0094 (15)	0.0126 (15)
C6	0.0214 (16)	0.0136 (14)	0.035 (2)	−0.0003 (12)	0.0100 (15)	0.0068 (14)
C7	0.0257 (16)	0.0146 (13)	0.0127 (14)	−0.0069 (12)	0.0013 (12)	0.0041 (11)
C8	0.0237 (16)	0.0138 (13)	0.0150 (15)	−0.0029 (12)	0.0024 (12)	0.0037 (11)
C9	0.0280 (17)	0.0181 (14)	0.0154 (15)	−0.0044 (13)	0.0055 (13)	0.0054 (12)
C10	0.0248 (17)	0.0261 (17)	0.0211 (17)	−0.0098 (14)	0.0033 (14)	0.0076 (14)
C11	0.0210 (17)	0.0272 (18)	0.030 (2)	−0.0083 (14)	−0.0032 (14)	0.0125 (16)
C12	0.0238 (17)	0.0245 (16)	0.0217 (17)	−0.0102 (14)	−0.0055 (14)	0.0107 (14)
C13	0.0234 (17)	0.0226 (16)	0.0281 (19)	0.0018 (14)	0.0070 (15)	0.0100 (15)
C14	0.0262 (19)	0.039 (2)	0.040 (2)	−0.0144 (17)	−0.0116 (17)	0.025 (2)
C15	0.0154 (14)	0.0140 (13)	0.0248 (17)	0.0000 (11)	0.0043 (13)	0.0036 (12)
C16	0.0201 (16)	0.0214 (15)	0.0216 (17)	−0.0060 (13)	0.0092 (13)	−0.0040 (13)
C17	0.0255 (18)	0.040 (2)	0.0127 (16)	−0.0054 (16)	0.0041 (13)	0.0028 (15)
C18	0.033 (2)	0.093 (4)	0.0161 (19)	−0.002 (3)	0.0097 (17)	0.016 (2)
C19	0.029 (2)	0.096 (5)	0.024 (2)	−0.003 (3)	0.0153 (18)	0.006 (3)
C20	0.0187 (18)	0.051 (3)	0.031 (2)	0.0007 (18)	0.0075 (16)	−0.007 (2)
C21	0.0205 (17)	0.0224 (16)	0.032 (2)	−0.0028 (13)	0.0060 (15)	−0.0046 (15)
C22	0.028 (2)	0.048 (3)	0.0192 (18)	−0.0027 (18)	0.0010 (15)	0.0124 (18)
C23	0.025 (2)	0.028 (2)	0.065 (3)	0.0054 (16)	0.007 (2)	0.016 (2)

### Chlorido(2,6-dimethylphenyl isocyanide)[N'-(2,6-dimethylphenyl)-N-(pyridin-2-yl)carbamimidoyl]platinum(II) Geometric parameters (Å, º)

**Table:**

Pt1—C15	1.910 (3)	C11—C12	1.398 (5)
Pt1—C1	1.979 (3)	C11—H11	0.9500
Pt1—N1	2.048 (3)	C12—C14	1.504 (5)
Pt1—Cl1	2.3709 (9)	C13—H13A	0.9800
N1—C2	1.340 (5)	C13—H13B	0.9800
N1—C6	1.355 (4)	C13—H13C	0.9800
N2—C2	1.350 (4)	C13—H13D	0.9800
N2—C1	1.404 (4)	C13—H13E	0.9800
N2—H2	0.80 (6)	C13—H13F	0.9800
N3—C1	1.291 (4)	C14—H14A	0.9800
N3—C7	1.413 (4)	C14—H14B	0.9800
N4—C15	1.157 (5)	C14—H14C	0.9800
N4—C16	1.394 (4)	C16—C21	1.396 (6)
C2—C3	1.405 (5)	C16—C17	1.398 (6)
C3—C4	1.377 (5)	C17—C18	1.394 (6)
C3—H3	0.9500	C17—C22	1.505 (6)
C4—C5	1.401 (6)	C18—C19	1.387 (8)
C4—H4	0.9500	C18—H18	0.9500
C5—C6	1.369 (5)	C19—C20	1.382 (8)
C5—H5	0.9500	C19—H19	0.9500
C6—H6	0.9500	C20—C21	1.394 (6)
C7—C12	1.401 (5)	C20—H20	0.9500
C7—C8	1.408 (5)	C21—C23	1.496 (7)
C8—C9	1.399 (5)	C22—H22A	0.9800
C8—C13	1.506 (5)	C22—H22B	0.9800
C9—C10	1.390 (6)	C22—H22C	0.9800
C9—H9	0.9500	C23—H23A	0.9800
C10—C11	1.389 (5)	C23—H23B	0.9800
C10—H10	0.9500	C23—H23C	0.9800
			
C15—Pt1—C1	98.97 (14)	C11—C12—C14	120.3 (4)
C15—Pt1—N1	178.85 (14)	C7—C12—C14	120.7 (3)
C1—Pt1—N1	80.71 (12)	C8—C13—H13A	109.5
C15—Pt1—Cl1	85.69 (10)	C8—C13—H13B	109.5
C1—Pt1—Cl1	175.33 (10)	H13A—C13—H13B	109.5
N1—Pt1—Cl1	94.63 (9)	C8—C13—H13C	109.5
C2—N1—C6	119.7 (3)	H13A—C13—H13C	109.5
C2—N1—Pt1	113.5 (2)	H13B—C13—H13C	109.5
C6—N1—Pt1	126.8 (3)	H13D—C13—H13E	109.5
C2—N2—C1	119.2 (3)	H13D—C13—H13F	109.5
C2—N2—H2	125 (4)	H13E—C13—H13F	109.5
C1—N2—H2	115 (4)	C12—C14—H14A	109.5
C1—N3—C7	123.1 (3)	C12—C14—H14B	109.5
C15—N4—C16	166.0 (4)	H14A—C14—H14B	109.5
N3—C1—N2	114.0 (3)	C12—C14—H14C	109.5
N3—C1—Pt1	134.9 (3)	H14A—C14—H14C	109.5
N2—C1—Pt1	111.2 (2)	H14B—C14—H14C	109.5
N1—C2—N2	115.2 (3)	N4—C15—Pt1	171.2 (3)
N1—C2—C3	121.3 (3)	N4—C16—C21	117.0 (4)
N2—C2—C3	123.4 (3)	N4—C16—C17	118.8 (4)
C4—C3—C2	118.6 (3)	C21—C16—C17	124.2 (4)
C4—C3—H3	120.7	C18—C17—C16	116.4 (4)
C2—C3—H3	120.7	C18—C17—C22	121.9 (4)
C3—C4—C5	119.7 (3)	C16—C17—C22	121.7 (3)
C3—C4—H4	120.2	C19—C18—C17	120.8 (5)
C5—C4—H4	120.2	C19—C18—H18	119.6
C6—C5—C4	118.7 (3)	C17—C18—H18	119.6
C6—C5—H5	120.6	C20—C19—C18	121.3 (4)
C4—C5—H5	120.6	C20—C19—H19	119.4
N1—C6—C5	122.0 (4)	C18—C19—H19	119.4
N1—C6—H6	119.0	C19—C20—C21	120.2 (4)
C5—C6—H6	119.0	C19—C20—H20	119.9
C12—C7—C8	121.0 (3)	C21—C20—H20	119.9
C12—C7—N3	119.4 (3)	C20—C21—C16	117.2 (4)
C8—C7—N3	119.2 (3)	C20—C21—C23	122.2 (4)
C9—C8—C7	118.4 (3)	C16—C21—C23	120.6 (4)
C9—C8—C13	122.2 (3)	C17—C22—H22A	109.5
C7—C8—C13	119.4 (3)	C17—C22—H22B	109.5
C10—C9—C8	120.9 (3)	H22A—C22—H22B	109.5
C10—C9—H9	119.5	C17—C22—H22C	109.5
C8—C9—H9	119.5	H22A—C22—H22C	109.5
C11—C10—C9	120.0 (3)	H22B—C22—H22C	109.5
C11—C10—H10	120.0	C21—C23—H23A	109.5
C9—C10—H10	120.0	C21—C23—H23B	109.5
C10—C11—C12	120.5 (4)	H23A—C23—H23B	109.5
C10—C11—H11	119.7	C21—C23—H23C	109.5
C12—C11—H11	119.7	H23A—C23—H23C	109.5
C11—C12—C7	119.0 (3)	H23B—C23—H23C	109.5
			
C7—N3—C1—N2	172.0 (3)	C8—C9—C10—C11	1.8 (6)
C7—N3—C1—Pt1	−8.2 (6)	C9—C10—C11—C12	−3.1 (6)
C2—N2—C1—N3	−175.3 (3)	C10—C11—C12—C7	1.2 (6)
C2—N2—C1—Pt1	4.9 (4)	C10—C11—C12—C14	−179.6 (4)
C6—N1—C2—N2	−179.5 (3)	C8—C7—C12—C11	2.0 (6)
Pt1—N1—C2—N2	2.6 (4)	N3—C7—C12—C11	−170.8 (3)
C6—N1—C2—C3	2.1 (5)	C8—C7—C12—C14	−177.2 (4)
Pt1—N1—C2—C3	−175.9 (3)	N3—C7—C12—C14	10.0 (5)
C1—N2—C2—N1	−5.0 (5)	C15—N4—C16—C21	9.9 (16)
C1—N2—C2—C3	173.4 (3)	C15—N4—C16—C17	−169.7 (13)
N1—C2—C3—C4	−0.5 (5)	N4—C16—C17—C18	177.9 (4)
N2—C2—C3—C4	−178.8 (4)	C21—C16—C17—C18	−1.6 (6)
C2—C3—C4—C5	−1.0 (6)	N4—C16—C17—C22	−3.0 (6)
C3—C4—C5—C6	1.0 (6)	C21—C16—C17—C22	177.4 (4)
C2—N1—C6—C5	−2.1 (6)	C16—C17—C18—C19	0.7 (8)
Pt1—N1—C6—C5	175.6 (3)	C22—C17—C18—C19	−178.3 (5)
C4—C5—C6—N1	0.6 (6)	C17—C18—C19—C20	0.8 (9)
C1—N3—C7—C12	−80.3 (5)	C18—C19—C20—C21	−1.5 (8)
C1—N3—C7—C8	106.8 (4)	C19—C20—C21—C16	0.7 (7)
C12—C7—C8—C9	−3.3 (5)	C19—C20—C21—C23	−179.2 (5)
N3—C7—C8—C9	169.5 (3)	N4—C16—C21—C20	−178.6 (3)
C12—C7—C8—C13	175.0 (3)	C17—C16—C21—C20	1.0 (6)
N3—C7—C8—C13	−12.2 (5)	N4—C16—C21—C23	1.2 (6)
C7—C8—C9—C10	1.4 (5)	C17—C16—C21—C23	−179.2 (4)
C13—C8—C9—C10	−176.9 (3)		

### Chlorido(2,6-dimethylphenyl isocyanide)[N'-(2,6-dimethylphenyl)-N-(pyridin-2-yl)carbamimidoyl]platinum(II) Hydrogen-bond geometry (Å, º)

*Cg*3 is the centroid of the C7–C12 benzene ring attached to the N3 atom.

**Table:**

*D*—H···*A*	*D*—H	H···*A*	*D*···*A*	*D*—H···*A*
N2—H2···N3^i^	0.80 (6)	2.15 (6)	2.943 (4)	173 (6)
C6—H6···Cl1	0.95	2.70	3.308 (4)	123
C13—H13*A*···N3	0.98	2.36	2.848 (5)	110
C22—H22*B*···Cl1^ii^	0.98	2.78	3.660 (5)	150
C3—H3···*Cg*3^i^	0.95	2.99	3.898 (4)	162

Symmetry codes: (i) −*x*+1, −*y*+1, −*z*+1; (ii) −*x*+1, −*y*, −*z*.
